# Long-Term Perspectives on Chronic Rhinosinusitis with Nasal Polyps: Evaluating Recurrence Rates after Functional Endoscopic Sinus Surgery in the Biologics Era—A 5-Year Follow-Up Study

**DOI:** 10.3390/jpm14030297

**Published:** 2024-03-10

**Authors:** Carlo Cavaliere, Simonetta Masieri, Elona Begvarfaj, Antonella Loperfido, Silvia Baroncelli, Francesca Cascone, Andrea Ciofalo

**Affiliations:** 1 Department of Sense Organs, Sapienza University, 00161 Rome, Italy; 2 Department of Oral and Maxillofacial Sciences, Sapienza University, 00161 Rome, Italy; 3 Department of Psychiatry, University of Campania “Luigi Vanvitelli”, 80138 Naples, Italy; 4 Otolaryngology Unit, San Camillo Forlanini Hospital, 00152 Rome, Italy; 5 National Center for Global Health, Istituto Superiore di Sanità, 00161 Rome, Italy

**Keywords:** chronic rhinosinusitis, CRSwNP, nasal polyps, FESS, monoclonal antibodies

## Abstract

Introduction: Chronic rhinosinusitis with nasal polyps (CRSwNP) is an inflammatory disease with multifactorial etiopathogenesis. This study investigated the recurrence rate and risk factors predicting recurrence in patients subjected to Functional Endoscopic Sinus Surgery (FESS) for CRSwNP. Methods: Patients affected by CRSwNP who underwent FESS between January 2015 and March 2020 were enrolled. The recurrence rate and the influence of risk factors were assessed. Results: A total of 154 patients were included, 100 males and 54 females, aged 14–82 years (mean age 51.96 ± 16.27; median 52 years). Of 154 patients, 28 presented CRSwNP recurrence in a follow-up period ranging from 6 months to 69 months, with a recurrence rate of 18.2%. The recurrence rate was higher in patients aged between 31 and 50 years and between 51 and 70 years at the time of surgery than in those aged between 14 and 30 years and over 70 years. Furthermore, most patients with recurrence were men (61%), while 39% were women. A higher recurrence rate was observed between non-smokers (50%) and ex-smokers (36%), while only 14% declared themselves habitual smokers. Only four subjects (14%) had a positive family history of CRSwNP. Conclusion: To date, no specific biomarkers have been identified in order to determine the appropriate therapy for the patients affected by CRSwNP. Based on our results, we suggest that it is necessary for an accurate assessment of the CRSwNP patients to identify which phenotype/endotype each subject manifests based on medical history, endoscopy, computed tomography, and a laboratory evaluation.

## 1. Introduction

Chronic rhinosinusitis (CRS) with nasal polyps (wNP) is a heterogeneous inflammatory disease characterized by the presence, for at least 12 weeks, of two or more symptoms, one of which should be either nasal blockage/obstruction/congestion or nasal discharge (anterior/posterior nasal drip), ±facial pain/pressure, ±hyposmia/anosmia, with bilateral, endoscopic or radiological evidence of nasal polyposis in middle meatus [1,2]. CRSwNP is a topic of specific interest because it denotes a diagnosis with quite a few clinical categories, including allergic fungal rhinosinusitis (AFRS), cystic fibrosis, and CRSwNP not otherwise specified [1].

CRSwNP is mainly characterized by inflammation of the mucosa of the nose and paranasal sinuses and tissue remodeling. Environmental and host genetic factors interact over time to trigger one or more pathways (endotypes) of chronic inflammation that lead to the clinical presentation of CRSwNP (phenotype) [3,4].

In particular, the most recent international guidelines subdivide chronic rhinosinusitis (CRS) into different inflammatory endotypes characterized by specific pathobiological mechanisms, which may vary over time and among different sinonasal anatomical sites in individual patients.

Endotyping, based on cluster analysis of inflammatory biomarkers, has shown a wide distinction between type 2 (eosinophilic) and non-type 2 (non-eosinophilic) CRS inflammation profiles. This is because each inflammatory profile is characterized by distinct immune cells, inflammatory mediators, and physiological functions. Specifically, while type 2 CRS typically shows eosinophil-dominant inflammation, non-type 2 CRS is characterized by neutrophilic inflammation in the nasal mucosa. Non-type 2 endotype includes both type 1 and type 3 inflammatory pathways.

Type 1 inflammatory response is characterized by T helper 1 (Th1) cells, which mature in response to an interferon gamma (IFN-γ) and interleukin (IL)-12 environment and form IFN-γ and IL-2. IFN-γ and IL-12 typically act against viral pathogens.

The type 2 response includes other cytokines such as IL-4, IL-13, and IL-5, which regulate tissue regeneration after injury, protect against parasitic helminth infections, and are also typically associated with atopic and allergic disorders, such as allergic rhinoconjunctivitis, food allergy, and eosinophilic esophagitis (EoE).

Finally, the type 3 inflammation pathway is mediated by T helper 17 (Th17) cells, which mature in response to Transforming Growth Factor β (TGF-β) and IL-6. Type 3 cytokines include IL-17A and IL-22 and have targeted immunological effects against extracellular bacteria and fungi [1].

While the type 1 pathway is classically reported in patients affected by chronic rhinosinusitis without nasal polyps (CRSsNP) and the type 3 inflammatory profile is dominant in Asian patients with CRSwNP, it is estimated that between 83% and 91% of Western patients affected by CRSwNP manifest a type 2 inflammation [5,6].

This inflammatory endotype is characterized by specific innate and adaptative immunity mediators, such as Group 2 Innate Lymphoid Cells and Th2 lymphocytes, and, as mentioned, a peculiar cytokine pattern: IL-4, IL-13, and IL-5 [5,6,7,8,9]. The recruiting and chronic activation of inflammatory cells, such as eosinophils, basophils, and mastocytes, lead to specific sinonasal symptoms and tissue remodeling, with nasal polyps formation [10,11]. Other conditions, such as bronchial asthma, atopic dermatitis (AD), and aspirin-exacerbated respiratory disease (AERD, also called Samter’s triad) share the same inflammatory subset, representing common comorbidities of CRSwNP [10,11].

Recent studies have assessed the associations between clinical features of CRS and endotypes, finding that the presence of loss of smell is more strongly associated with type 2 inflammation; in contrast, the presence of purulent nasal discharge is mainly related to non-type 2 inflammation. Thus, endotyping highlighted the complexity of this inflammatory condition and some patients with CRS may even express a mix of two or more inflammatory endotypes [1].

First-line therapeutic options currently available for CRSwNP are represented by nasal corticosteroids [12,13,14] and saline rinses with or without systemic steroids [15], followed by a less or more extensive surgical approach for patients unresponsive to medical intervention [16,17]. The most used surgical approach for this condition is FESS (Functional Endoscopic Sinus Surgery), which involves the removal of inflammatory tissue and the opening of the sinus ostia to treat CRS and has the main advantage of being less invasive than other surgical interventions. However, these surgical strategies often fail to provide adequate control of symptoms or recurrences, because they do not consider the underlying pathophysiological mechanisms of such a heterogeneous disease and its comorbidities [18,19,20]. Recent studies have demonstrated that greater asthma severity has been related to worse radiological evidence of CRS [21]. On the other hand, pharmacological and surgical treatment of CRS in patients affected with asthma can decrease asthmatic symptoms and exacerbations [22].

Although CRSwNP affects approximately 3–6% of the general population [23], based on symptoms and endoscopic evaluation, it is burdened by high healthcare costs [24], due to the high rate of recurrence after surgery, and the need for multiple revision surgeries and several courses of oral corticosteroids (OCS) [25]. Moreover, despite optimal medical therapy, the low quality of life (QoL) negatively impacts patient productivity, increasing indirect costs [26]. In this regard, several authors have highlighted that CRSwNP negatively impacts QoL in a manner comparable to other debilitating chronic diseases such as diabetes, chronic obstructive pulmonary disease (COPD), and congestive heart failure [27,28]. Furthermore, from the patient’s perspective, how CRS affects daily life is much more important than the results of medical investigations such as CT scans [29]. Interestingly, further authors have even found an association between CRS and an increased incidence of anxiety and depression [30].

During the last few years, monoclonal antibodies (MAbs), already indicated as add-on therapy for severe asthma, have been successfully introduced as a treatment strategy for CRSwNP [31,32,33]. Considering the high costs of this class of drugs that could potentially be a “lifetime” therapy [34], it is mandatory to evaluate which comorbidities and markers (anamnestic, biochemical, instrumental, and histological parameters) could be related to a higher recurrence rate of the CRSwNP and might represent useful indicators for tracing CRSwNP endotype, consistent with the recent chronic rhinosinusitis classification.

The aim of this study has been the assessment of the CRSwNP recurrence rate after FESS and the valuation of recurrence risk factors.

## 2. Materials and Methods

This is a retrospective, observational study covering the period from January 2015 to March 2020. It involved all the patients affected by CRSwNP who underwent FESS in the Otorhinolaryngology Clinic of Policlinico Umberto I in Rome, Italy. 

A total of 154 patients were enrolled. Patients with concomitant cystic fibrosis, congenital or acquired immunodeficiencies, congenital mucociliary clearance disorders, sino-nasal fungal infections, systemic vasculitis or granulomatosis, and cocaine addiction were excluded. Demographics, smoking habits, heritability for CRSwNP, comorbidities (asthma, AERD, non-steroidal anti-inflammatory drugs (NSAID) intolerance, allergy, gastroesophageal reflux disease (GERD)), anatomic nasal variations, hyposmia/anosmia, peripheral blood eosinophils and basophils count, neutrophil-to-lymphocyte ratio (NLR), computed tomography (CT) of the paranasal sinuses, and previous nasal surgeries were investigated for each patient. All comorbidities were not self-reported but confirmed by medical reports. The patients with a diagnosis of asthma in their personal history underwent a pneumological evaluation by a specialist before the surgery. The presence of hyposmia/anosmia was investigated by olfactory tests (Sniffin’ Sticks, Burghart, Germany). The nasal function was evaluated using anterior rhinomanometry. A CT scan of paranasal sinuses was available in 107 patients, and a Lund–Mackay Score was calculated. This study was approved by the EC Lazio 1 n° 411, and informed consent was obtained from all patients included in this study.

### Statistical Analysis

Statistical analysis was performed using descriptive statistics, mean, and standard deviation (SD) for the quantitative variables, and absolute and percentage frequencies for the categorical ones. The results were summarized in graphs and tables. Data are described as mean (±SD) or number (% of the population). Differences between groups for quantitative variables were assessed by *t*-test, for qualitative variables by Chi-square test or Fisher’s test, if modalities with frequency < 5 were present. Survival to recurrence was presented using the Kaplan–Meier curve, and the risk of recurrence was assessed using a univariate and multivariate logistic model. A *p*-value < 0.05 was considered statistically significant. Statistical analysis was performed using STATA software, version 13 (StataCorp Release 13. College Station, TX, USA).

## 3. Results

Between January 2015 and March 2020, approximately 300 patients underwent FESS for CRSwNP in our department. A systematic analysis of medical records was conducted, and patients were selected in accordance with the exclusion criteria.

About 200 patients met the inclusion criteria, and follow-up was assessed in 154 cases. The recurrence of postoperative CRSwNP was diagnosed by evidence of bilateral nasal polyps on endoscopy and CT imaging along with the presence of symptoms as defined by the European Position Paper on Rhinosinusitis and Nasal Polyps (EPOS) [1,2]. 

A total of 154 patients were included, 100 males and 54 females, aged 14–82 years (mean age 51.96 ± 16.27; median 52 years). Characteristics of the cohort are described in Table 1.

About half of the cohort, 72 subjects (47%), were in the normal weight range, 58 subjects (38%) were overweight, 20 (12%) were obese, and only 4 patients (3%) were underweight. Regarding smoking habits, 58% of patients were non-smokers, 14% were habitual smokers, and 29% reported a previous smoking habit.

Heritability, an assessment based on nasal polyposis diagnosis in a first-degree relative, was found in only 12% of cases.

As for the comorbidities, bronchial asthma was the most common disease associated with CRSwNP (16% of cases), GERD, and intolerance to NSAIDs and aspirin (ASA–NSAID intolerance) affected 10% of the cohort. Also, 38% of patients reported at least one allergic disease in their medical history. In particular, “seasonal” allergies (pollinosis) were found in 21% of cases, “perennial” allergies (dust mites, animal hair) in 9% and mixed forms in 8%.

Severe symptoms before surgery, such as nasal obstruction, hyposmia, and/or anosmia, were detected in 56% and 40% of patients, respectively.

About half of the patients presented septal deviation and turbinate hypertrophy (51% and 53%, respectively).

The patients with no history of previous surgery were 118 (77%), while 36 (23%) reported at least one surgical intervention for nasal polyposis.

**Figure 1:**
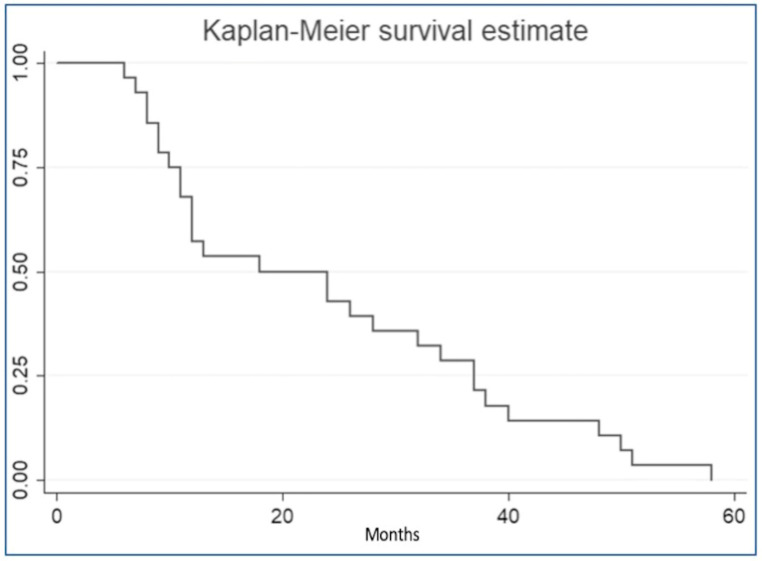
Kaplan–Meier survival curve of CRSwNP recurrence.

Of 154 patients, 28 presented CRSwNP recurrence in a follow-up period ranging from 6 months to 69 months, with a recurrence rate of 18.2%. In 9 patients (32%), recurrence occurred within 12 months after surgery, in 7 patients (25%) between 12 and 24 months, in 4 patients (14%) between 25 and 36 months, in 5 patients (18%) between 37 and 48 months and in 3 patients (11%) between 49 and 60 months. The average time of recurrence was 24.04 (±15.88) months. The median survival time (to recurrence) was 18 months (Figure 1).

The rate of recurrence was higher in patients aged between 31 and 50 years (43%) and between 51 and 70 years (36%) at the time of surgery than in those aged between 14 and 30 years (10.5%) and over 70 years (10.5%). Furthermore, most patients with recurrence were men (61%), while 39% were women. Regarding the Body Mass Index (BMI), out of 28 patients with recurrence, 1 (4%) was underweight (BMI: 16–18.5), 17 (61%) were in the normal weight range (BMI: 18.6–25), 9 (32%) were overweight (BMI: 25.1–30), and 1 (4%) was affected by mild obesity (BMI: 30.1–35).

A higher recurrence rate was observed between non-smokers (50%) and ex-smokers (36%), while only 14% declared themselves habitual smokers. Only four subjects (14%) had a positive family history of CRSwNP.

Regarding comorbidities, GERD affected 1 subject (4%), bronchial asthma affected 12 subjects (43%), and AERD affected 5 subjects (18%). Allergies were reported by 57% of patients, of which 36% were seasonal allergies (pollinosis), 14% were perennial allergies (dust mites, animal hair), and 7% were mixed forms, both seasonal and perennial. No history of allergy was reported by 43% of patients. ASA–NSAID intolerance affected 8 of the relapsed patients (29%).

Clinically relevant nasal septal deviation, identified by preoperative anterior rhinoscopy, was diagnosed in fourteen patients (50%) with CRSwNP recurrence. Turbinate hypertrophy was found in eleven patients (39%). Ten subjects (36%) presented preoperative hyposmia and/or anosmia, and eleven (39%) presented nasal obstruction in anterior rhinomanometry.

Among patients with CRSwNP recurrence, twelve of them (42%) reported no previous surgery, eight (29%) had one previous surgery, and eight (29%) had more than one.

The Lund–Mackay CT score was calculated in 107 patients out of 154. In the group of patients with CRSwNP recurrence, the mean Lund–Mackay score was 16.60 (±4.44), while in the non-recurrence group, it was 11.38 (±5.00).

The peripheral blood eosinophilic mean count was 0.53 (±0.39) (×10^9^/L) in the recurrence group, compared with 0.34 (±0.28) (×10^9^/L) in the non-recurrence group.

The peripheral blood basophils mean count was 0.04 (±0.02) (×10^9^/L) in the recurrence group and 0.05 (±0.03) (×10^9^/L) in the non-recurrence group. The mean NLR (neutrophil-to-lymphocyte ratio) was 1.84 (±0.62) in the recurrence group and 2.34 (±1.02) in the non-recurrence group (Table 2).

The univariate statistical analysis highlighted a strong association between the recurrence of nasal polyposis and comorbidities such as bronchial asthma (OR: 6.5—*p* < 0.001), AERD (OR: 13.5—*p* = 0.003), and ASA-NSAID intolerance (OR: 5.9—*p* = 0.001). Furthermore, a history of previous surgery resulted strongly related to recurrence: an augmented risk was assessed for patients with at least one previous surgery for CRSwNP (1 previous surgery OR: 4.7—*p* = 0.004, >1 previous surgery OR: 14.1—*p* < 0.001). Moreover, peripheral blood eosinophilia (OR: 5.1—*p* = 0.014) is associated with an increased risk of recurrence, while the detection of a high NLR seems to predict a reduced probability of recurrence (OR: 0.5—*p* = 0.018). The Lund–Mackay (L-M) score calculated on preoperative CT images was found to be a good predictor of recurrence (OR: 1.2—*p* < 0.001). In particular, a cut-off value of ≥9 seems to progressively increase the risk of recurrence (*p* = 0.029).

Both age and BMI were not statistically associated with the recurrence of CRSwNP, although the age range of 40–60 years might be at higher risk of recurrence (OR: 1.4—*p* = 0.468).

Multivariate logistic regression was performed to identify independent predictors of CRSwNP recurrence. Multivariate statistical analysis showed that only the preoperative CT L-M score was found to be associated with CRSwNP recurrence (OR: 1.3—*p*-value = 0.08).

## 4. Discussion

CRSwNP is a disease burdened by a high rate of recurrence despite an optimal therapeutic approach [18,24]. The “failure” of the current treatments in the long-term control of the disease involves a high impact of CRSwNP, both in terms of reduced QoL of the individual patient, and of the high socio-economic costs [35,36]. It is estimated that the direct costs, including healthcare costs for the treatment of the disease prior to surgery, amount between USD 1547 and USD 23,000 per patient/year, and are even higher in the case of recurrent polyposis after surgery [36,37]. The indirect costs are related to reduced individual work productivity due to the negative impact of symptoms since 85% of patients affected belong to the working-age population (18–65 years) [36]. In light of the recent introduction of new therapeutic possibilities represented by biological drugs, it is beneficial to understand the factors associated with polyps recurrence after surgery to provide the best treatment to the patient. “Biologics”, which selectively target the type 2 inflammatory cascade by binding to specific interleukins such as IL-4, IL-5, and IL-13 or their respective receptors, is a reliable treatment option for all those CRSwNP patients who experience failure of conventional therapy during follow-up [5,34].

The main purpose of our study was to investigate the clinical, biochemical, and instrumental parameters that could be potential predictors of recurrence and could be helpful in defining the CRSwNP endotype, allowing monitoring with a closer follow-up with those patients undergoing surgery who are at greater risk of polyps’ relapse and become eligible for MAbs therapy. We purposefully choose to include in this study solely objective and easily assessable parameters, because PROMs (patient-reported outcome measures) in a retrospective real-life study could lead to unreliable results in the calculation. The identification of consistent biomarkers with real-life practice methods may support clinicians in deciding adequate and personalized therapy schedules for patients with CRSwNP.

The CRSwNP recurrence rate in our cohort was 18.2% (28 out of 154 patients); in the literature, the recurrence rate varies from 20% up to 70% depending on the follow-up time taken into consideration and what is meant by recurrence [18,19,20]. In this case, the strength of our data is linked to the fact that this is a single-center study where the patients were all operated by three surgeons who share the same surgical training without any patients undergoing polypectomy. Furthermore, the high rate of patients undergoing the first FESS (77%) allowed us to observe a population with a less severe or, in any case, “younger” disease compared to the population with recurrent severe disease. This demonstrates how the surgical approach, although it represents the current standard of care along with corticosteroid therapy, cannot be considered a definitive treatment, especially in those patients who relapse within a short time, specifically the 12.98% of patients who relapse within 36 months of surgery.

The comorbidities strongly associated with the disease recurrence, according to our study and in agreement with the evidence reported in the literature are bronchial asthma (OR: 6.5—*p* < 0.001), AERD (OR: 13.5—*p* = 0.003), and the NSAID intolerance (OR: 5.9—*p* = 0.001) [18,38,39].

Therefore, these comorbidities could represent clinical indicators that, along with biochemical or histological parameters, would allow the identification of patients with CRSwNP type 2 inflammatory signature, with an increased risk of recurrence after surgery [38]. Patients with CRSwNP and asthma present an augmented risk of disease recurrence, as well as greater severity of CRS and lesser control of asthma. The severity and lack of control of asthmatic symptoms have been associated with a higher endoscopic and radiological score of nasal polyposis, and better control of CRS can decrease asthmatic symptoms and exacerbations [21]. This suggests that we should always perform a multidisciplinary evaluation of the patients affected by inflammatory pathologies of upper and lower airways: an ENT evaluation of the patients with asthma, and the evaluation of the pulmonary function of the patients with CRSwNP.

The peripheral blood eosinophil count was higher in the group of patients who relapsed (0.53 ± 0.39 × 109/L vs. 0.34 ± 0.28 × 109/L). On the other hand, either peripheral blood basophil count or NLR was lower in the patients with nasal polyps’ recurrence, inconsistent with data reported by other authors [40].

In accordance with current evidence in the literature [41], and also the surgical history of the individual patient, the intention to have at least one surgery for CRSwNP performed in the past seems to be prognostically relevant (OR: 14.1—*p* < 0.001) with increased risk of recurrence. A higher number of prior surgeries for CRSwNP may reflect a more severe underlying inflammatory endotype or may be due to poor patient compliance with post-surgery medical therapy or follow-up visits.

The mean time from surgery to the first CRSwNP recurrence in our subjects was 22 months, emphasizing the concept that the relapse of the disease is not immediate but usually happens after a period of time. Consequently, the management of these patients should be based on long-term follow-up, through timely re-evaluations based on the endoscopic examination as well as on the severity of symptoms assessment.

The finding of nasal anatomical anomalies on anterior rhinoscopy during the ENT physical examination, such as septal deviation or turbinate hypertrophy, is not related to an increased risk of recurrence (*p* = 0.882 and *p* = 0.125); thus, it is likely that upper respiratory tract alterations, even if severe, are not pathogenetically associated with nasal polyposis and, in particular, with the recurrence of the disease. Even the presence of preoperative hyposmia/anosmia does not appear to be related to a higher frequency of recurrence (*p* = 0.701), as olfactory function alterations may be due to the inflammatory state of the mucosa, even in the absence of polyposis.

Among the preoperative instrumental investigations, the Lund–Mackay score calculated on a CT scan of the paranasal sinuses was found to be able to identify patients with a higher risk of recurrence. In particular, in our study, and consistent with other data [42,43], the mean L-M score values were higher in the group of patients who relapsed (16.60 ± 4.44 vs. 11.38 ± 5.00; *p* < 0.001). Moreover, it was possible to calculate a statistically significant cut-off value = 9, beyond which the likelihood of recurrence seems to be progressively increased (*p* = 0.029). The study of Ching-Lung et al. [41], demonstrated that a Lund–Mackay score greater than 14 before the surgery would result in an increased recurrence rate. A CT score higher than 14 implies a substantial extension of the inflammation and a more complex pathology that likely would present a recurrence. The cut-off = 9 of our study permits us to calculate the probability of the recurrence even in cases that appear to be of lesser severity. Other studies with bigger cohorts of patients are necessary in order to demonstrate a prognostic value of this parameter. However, there is no consensus about the prognostic rate of CT scans, as other studies have shown no significant association between CT score and recurrence or revision surgery rate [44,45].

As for the demographic parameters, neither the age nor the gender of the patients was found to be statistically significant in causing recurrence (*p* = 0.226 and *p* = 0.608, respectively).

Additionally, there is no statistical significance of association between BMI and the risk of recurrence, in contrast to other studies that hypothesize an etiopathological correlation between CRS, metabolic syndrome, and visceral obesity due to the secretion of proinflammatory mediators, such as cytokines and adipokines from excess adipose tissue [46]. Concerning the smoking habit, which has an established correlation with the occurrence of CRS due to tobacco-induced bacterial biofilm formation [4] and the irritating and immunosuppressive effects of smoking on sinonasal mucosal cells [47], no significant correlation with recurrence was found (*p* = 0.551) in our study.

Heritability of nasal polyposis does not seem to play a prominent role in the onset of recurrence (*p* = 0.666), as reported previously by others [48]. All these findings could indicate that, unlike the pathogenetic moment of onset of the disease, recurrence is more influenced by the inflammatory endotype than a possible, specific but still unclear genetic predisposition (sex, heritability) or environmental factor, such as tobacco smoke.

Allergic disease, particularly rhinitis, does not seem to be a determining factor in the recurrence (*p* = 0.151), although it is still related to the onset of the disease [49]. However, determining the possible presence of allergy in the diagnostic work-up phase could be an important indicator of type 2 inflammation and help to trace a clinical/biochemical profile of the patient that can support the choice of integrated therapeutic options to the surgical approach [49,50].

Our study has some limitations, linked primarily to its retrospective nature and the impossibility of investigating parameters, such as IL-5, total IgE, and IL-4/IL-13, which are targets of the current therapy with Mabs [51,52,53,54].

It is known that the pathophysiology of CRSwNP is characterized by increased tissue levels of IL-5 and, currently, the therapeutic option represented by Mepolizumab, a monoclonal anti-IL5 antibody, is effective in nasal polyposis and it could be useful in preventing a possible recurrence of the disease and consequent revision surgery [33,55]. Dupilumab inhibits the dual signaling pathways of IL-4 and IL-13, which are key and central drivers of type 2 inflammation in CRSwNP [31,56]. Tissue IgE is also a key element in type 2 inflammation characterizing CRSwNP [57]. Specifically, IgE represents a target of Omalizumab therapy, which prevents IgE binding to the high-affinity IgE receptor (FcεRI) on the surface of basophils and mast cells. This Mab has also already been approved for the treatment of CRSwNP [32].

## 5. Conclusions

To date, no specific biomarkers have been identified in order to decide the appropriate therapy for the patients affected by CRSwNP. Based on our results, it is necessary to accurately assess the CRSwNP patients to identify which phenotype/endotype each subject manifests based on medical history, endoscopy, CT, and a laboratory evaluation. Furthermore, a multidisciplinary evaluation of the patient should be mandatory, especially for those comorbidities (asthma, AERD, NSAIDs intolerance), which, being an expression of type 2 inflammation, are closely associated with CRSwNP and the likelihood of its recurrence.

## Figures and Tables

**Table 1:** Population characteristics.

Characteristics		N (%)	Mean (±SD)
Age at Enrollment (years)			51.96 (±16.27)
Gender	Males	100 (65)	
	Females	54 (35)	
BMI	Underweight (16–18.5)	4 (3)	
	Normal Weight (18.6–25)	72 (47)	
	Overweight (25.1–30)	58 (38)	
	Obese (>30)	20 (12)	
Smoking Habits	Smokers	21 (14)	
	Ex-smokers	44 (29)	
	No Smokers	89 (58)	
Heritability of CRSwNP		18 (12)	
Comorbidities	Asthma	25 (16)	
	GERD	15 (10)	
	NSAID Intolerance	16 (10)	
Allergy	Seasonal Allergies	32 (21)	
	Perennial Allergies	14 (9)	
	Both Seasonal and Perennial Allergies	12 (8)	
Clinical Symptoms	Hyposmia/Anosmia	61 (40)	
	Nasal Obstruction	87 (56)	
Anterior Rhinoscopy	Nasal Septum Deviation	79 (51)	
	Turbinate Hypertrophy	81 (53)	
Previous Surgeries	0	118 (77)	
	≥1	36 (23)	

Preoperative characteristics of the surgical cohort with chronic rhinosinusitis with nasal polyps (N = 154). Data are described as mean (±SD) or number (% of the population). CRSwNP: chronic rhinosinusitis with nasal polyps.

**Table 2:** Group comparison.

	Recurrence		No Recurrence		*p*-Value
	Mean	SD	Mean	SD	
Lund–Mackay score	16.60	4.44	11.38	5.00	<0.001
Eosinophils (×10^9^/L)	0.53	0.39	0.34	0.28	0.003
Basophils (×10^9^/L)	0.04	0.02	0.05	0.03	0.401
NLR	1.84	0.62	2.34	1.02	0.015

Mean, standard deviation (SD), and *p*-value of Lund–Mackay score, peripheral blood eosinophils and basophils count, and neutrophils-to-lymphocytes ratio in the two groups of patients: recurrence group and no-recurrence group. NLR: neutrophil-to-lymphocyte ratio.

## Data Availability

The data that support the findings of this study are available from the corresponding author upon reasonable request.
